# Frequency of urinary incontinence and attitudes towards urinary incontinence of women in Mersin, Turkey

**DOI:** 10.12669/pjms.40.7.8134

**Published:** 2024-08

**Authors:** Nazife Akan, Behire Sancar

**Affiliations:** 1 Nazife Akan, Assistant Professor, Department of Nursing, Faculty of Health Sciences, Toros University, 45 Evler Campus, Mersin, Turkey; 2 Behire Sancar, Assistant Professor, Department of Nursing, Faculty of Health Sciences, Toros University, 45 Evler Campus, Mersin, Turkey

**Keywords:** Attitude, Urinary incontinence, Women’s health

## Abstract

**Objectives:**

Urinary incontinence (UI) is the involuntary loss of urine or the inability to control urination. The International Continence Society defines UI as any complaint of involuntary loss of urine. UI is a very important health problem that affects women’s quality of life. It can lead to a decrease in women’s self-confidence and prevent them from participating in social activities. This study was conducted to investigate the prevalence of UI in women living in Turkey, the factors affecting it, and women’s attitudes toward UI.

**Methods:**

This descriptive and cross-sectional study included 903 women who applied to 17 family health centers in Mersin, Turkey, between May 10^th^ to June 10^th^ 2019 for any reason. The data of the study were obtained using the descriptive information form and the Urinary Incontinence Attitude Scale (UIAS). Data analysis was performed with SPSS-21 using percentiles, mean±standard deviation, and Pearson chi-square tests.

**Results:**

According to the results obtained, 28.9% of the women had UI problems, more than half (54.6%) of the women with UI did not present to a health institution for this problem, and 54.8% did not receive any treatment. In addition, the frequency of experiencing UI was higher in those with a chronic disease (59.2%) than in those without any chronic disease, and the difference was significant.

**Conclusion:**

The demographic and fertility characteristics of the women influenced the frequency of experiencing UI. In addition, among the women participating in the study, those with negative attitudes toward UI experienced more UI problems. There should be greater efforts to raise awareness among women on this subject.

## INTRODUCTION

Involuntary urinary incontinence (UI) or the inability to control urination^1^ is a common urogenital health problem that impairs the quality of life of women. High fertility, large baby, cesarean sections (CS) history, pelvic operations, advanced age, obesity, chronic diseases, and genetic predisposition are among the factors that impair the functional structure of the pelvic floor.^2^,^3^ Women with UI are afraid of being noticed by others, they feel flawed, inadequate, and unattractive.^4^ Women with UI mostly do not want to visit a health institution because of embarrassment or perceiving the problem as a normal situation.^5^ Urinary incontinence rates have varied in studies conducted in different regions in Turkey.^2^ This study was conducted to evaluate the frequency of urinary incontinence in women living in the city of Mersin, which is located in the Eastern Mediterranean Region of Turkey, receives immigration and has a very high fertility rate, and women’s attitudes towards urinary incontinence.

## METHODS

The is a descriptive and correlational study. It included 903 women who visited 17 local family health centers in Mersin, for any reason between 10 May 10^th^ and June 10^th^ 2019. No sample selection method was used. Data were obtained with the Descriptive Information Form and the Urinary Incontinence Attitude Scale (UIAS). The descriptive information form, developed by the researchers in the light of the literature, consisted of a total of 35 questions aimed at evaluating the sociodemographic, fertility and UI status of women.^6^,^7^

### Ethical Approval:

It was obtained from the Publication Ethics Board of a university (dated April 25, 2019 and numbered 13). This study was conducted in accordance with the principles of the Declaration of Helsinki.

### Urinary Incontinence Attitude Scale (UIAS):

UIAS was developed by Henderson and Kashka in 1999 to determine adults’ attitudes towards urinary incontinence.^8^ In the validity and reliability study of the scale in Turkey, Gungor Uğurlucan F et al. and the Cronbach Alpha value of the scale was found to be 0.72. The UIAS is a 15-item questionnaire that measures attitudes towards UI. Each question has the four following response options: 1- Strongly disagree, 2- Disagree, 3- Agree, 4- Strongly agree. The total score is obtained by reverse coding the negative questions and summing the answers to all the questions. The total score ranges from 15 to 60 and there is no cut-off point. Positive approaches are measured by high scores.^5^

The Questionnaire used in the research were shared with women face to face, after they signed the informed consent form. It took approximately 15 minutes to fill out the forms.

### Inclusion and Exclusion Criteria:

Woman over the age of 18 and not having any communication problems, and those who were not agreeing to participate in the study and having communication problems were excluded.

### Statistical analysis:

The data analysis was performed with SPSS-21 using percentiles, mean±standard deviation, and Pearson’s chi-square tests. In the statistical evaluation, a p-value ≤0.05 was considered significant.

## RESULTS

**Fig.1:**
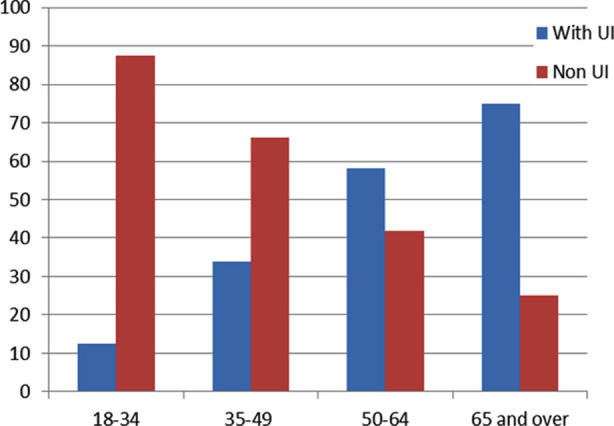
Frequency of UI in Women by Age.

In our study, 28.9% of the women had UI problems. The frequency of UI was higher in women aged 65 and over (75.0%) (Fig.1), women with a BMI of 30 and above (52.7%) (Fig.1). In the statistical analysis the difference between the groups was equally significant for all variables (p<0.001) Table-I.

**Fig.2:**
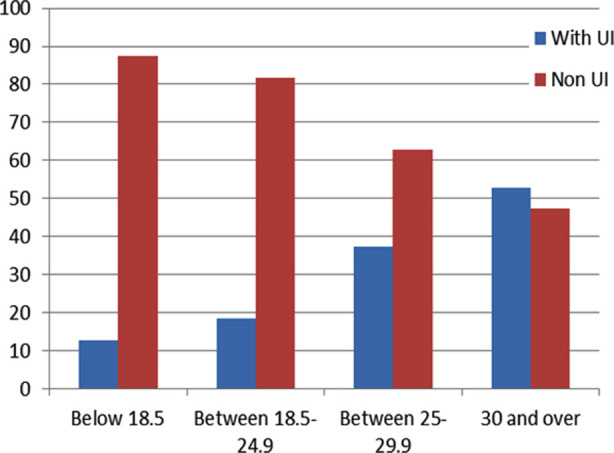
Frequency of UI in Women According to BMI.

**Fig.3:**
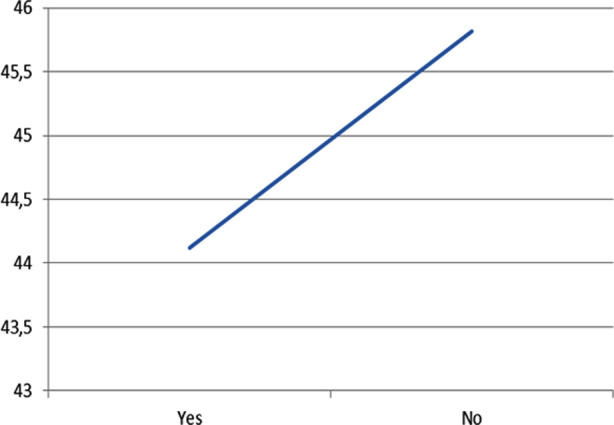
Comparison of UIAS scores of women according to chronic disease status (N: 903).

**Table-I:** Prevalence of UI by Demographic Characteristics of Women (N: 903)

Frequency of UI				

Demographic Characteristics of Women	With UI Frequency Percent (%)	Non UI Frequency Percent (%)	Total Frequency Percent (%)	*p
***Age group***				p<0.001
18-34	54(12.4)	380(87.6)	434(48.1)	
35-49	102(33.9)	199(66.1)	301(33.3)	
50-64	72(58.1)	52(41.9)	124(13.7)	
65 and over	33(75.0)	11(25.0)	44(4.9)	
***Level of education***				p<0.001
Primary	112(51.1)	107(48.9)	219(24.2)	
Secondary	85(29.8)	200(70.2)	285(31.6)	
Tertiary	43(12.9)	290(87.1)	333(36.9)	
***Occupation***				p<0.001
Health employee	10(13.0)	67(87.0)	77(8.5)	
Retired	8(44.4)	10(55.6)	18(2.0)	
Housewife	156(44.1)	198(55.9)	354(39.2)	
Other	87(19.2)	367(80.8)	454(50.3)	
***Marital status***				p<0.001
Single Married	24(9.3) 205(36.1)	233(90.7) 363(63.9)	257(28.5) 568(62.9)	
Divorced	32(41.0)	46(59.0)	78(8.6)	
***BMI***				p<0.001
Below 18.5	8(12.7)	55(87.3)	63(7.0)	
Between 18.5-24.9	79(18.4)	351(81.6)	430(47.6)	
Between 25-29.9	104(37.3)	175(62.7)	279(30.9)	
30 and over	69(52.7)	62(47.3	131(14.5)	

* Pearson chi-square or Fisher's exact tests were used according to their eye-expected frequencies.

In the study, the prevalence of UI was found to be higher in menopausal women (70.6%) and in women with seven or more pregnancies (67.7%), in women who gave birth longer than 24 hours (57.5%), women had postpartum urogenital tract infections (62.7%) and the difference was statistically significant (p<0.001). Table-II.

**Table-II:** Frequency of UI According to Fertility and Health Characteristics of Women (N: 903).

	Frequency of UI			

Some Characteristics of Women	With UI Frequency (%)	Non UI Frequency (%)	Total Frequency (%)	*p
***Menstrual status***			
Regular/Irregular	146(19.7)	594(80.3)	740(81.9)	p<0.001
In menopause	115(70.6)	48(29.4)	163(18.1)	
***Menopause duration (n: 163)***			
One year and less	4(40.0)	6(60.0)	10(6.1)	p=0.155
2-10 years	61(61.6)	38(38.4)	99(60.7)	
11-20 years	25(75.8)	8(24.2)	33(20.2)	
21 years and more	15(71.4)	6(28.6)	21(13.0)	
***Number of preg. (n: 610)***			
Between 1-3	126(29.6)	299(70.4)	425(69.7)	p<0.001
Between 4-6	87(56.5)	67(43.5)	154(25.2)	
7 and above	21(67.7)	10(32.3)	31(5.1)	
***Gb (n: 903)***			
Yes	226(38.7)	358(61.3)	584(64.7)	p<0.001
No	35(11.0)	284(89.0)	319(35.3)	
***Number of births (n: 584)***			
1-2	95(28.4)	239(71.6)	334(57.2)	p<0.001
3 and above	131(52.4)	119(47.6)	250(42.8)	
***Gb less than 2 years apart (n: 612)***			
Yes	93(52.5)	84(47.5)	177(28.9)	p<0.001
No	141(32.4)	294(67.6)	435(71.1	
***Type of Gb (n: 587)***			
VD	158(45.8)	187(54.2)	345(58.8)	p<0.001
CS	68(28.1)	174(71.9)	242(41.2)	
***Gb longer than 24 hours (n: 582)***			
Yes	50(57.5)	37(42.5)	87(14.9)	p<0.001
No	173(34.9)	322(65.1)	495(85.1)	
***Gb to a baby 3.500 g/more (n: 586)***			
Yes	125(47.7)	137(52.3)	262(44.7)	p<0.001
No	101(31.2)	223(68.8)	324(55.3)	
***Surgical suture if VD made (n: 345)***			
Yes	130(47.8)	142(52.2)	272(78.8)	p=0.418
No	31(42.5)	42(57.5)	73(21.2)	
***Anesthesia at CS (n: 242)***			
Spinal anesthesia	25(21.4)	92(78.6)	117(48.3)	p=0.012
General anesthesia	45(36.0)	80(64.0)	125(51.7)	
***Postpartum USI (n: 584)***			
Yes	69(62.7	41(37.3)	110(18.8)	p<0.001
No	154(32.5)	320(67.5)	474(81.2)	
***Urin/gen. tract inf. (n: 903)***			
Yes	173(37.2)	292(62.8)	465(51.5)	p<0.001
No	88(20.1)	350(79.9)	438(48.5)	
***Chronic illness (n: 903)***			
Yes	142(59.2)	98(40.8	240(26.6)	p<0.001
No	119(17.9)	544(82.1)	663(73.4)	
***Actions to prevent UI (n: 903)***			
Seeking a doctor	32(30.2)	74(69.8)	106(11.7)	p=0.814
Using medication	4(36.4)	7(63.6)	11(1.2)	
She does not know	225(28.6)	561(71.4)	786(87.1)	
***Exercise to prevent UI (n: 903)***			
She knows	82(42.9)	109(57.1)	191(21.2)	p<0.001
She does not know	179(25.1)	533(74.9)	712(78.8)	

Gb: Giving birth. preg: pregnancies. Urin/gen: Urinary/genital.

* Pearson chi-square and Fisher’s exact tests were used according to the expected frequencies.

Women in the 18-34 age group had the highest mean UIAS (45.92±5.77). Those without chronic disease had a higher mean UIAS (45.82±5.63) and the difference was statistically significant (p< 0.05.Table-III, Fig.1

**Table-III:** Comparison of UIAS Scores of Women According to Some Characteristics (N: 903*).

Some Characteristics of Women	UIAS Total Scores mean±SD	p	Some Characteristics of Women	UIAS Total Scores mean±SD	p
Age **		<0.05	BMI**		>0.05
18-34	45.92 ± 5.77^a^		Below 18.5	45.46 ± 5.67	
35-49	45.40 ± 6.00^ab^		Between 18.5-24.9	45.63 ± 5.80	
50-64	44.24 ± 5.40^bc^		Between 25-29.9	45.25 ± 6.33	
65 and over	42.58 ± 7.14^c^		30 and over	44.65 ± 5.43	
***Education*** **		<0.05	Gb to a baby 3.500g or more***		>0.05
Primary	44.25 ± 6.03^a^				
Secondary	45.07 ± 5.68^ab^		Yes	45.09 ± 6.13	
Tertiary	46.24 ± 5.83^b^		No	44.95 ± 5.50	
***Occupation*** **		>0.05	***Urin/gen. tract inf.*** ***		>0.05
Health employee	46.50 ± 6.11			45.46 ± 6.40	
Retired	44.55 ± 6.28		Yes	45.27 ± 5.36	
Housewife	44.79 ± 6.17		No		
Other	45.66 ± 5.62				
***Marital status*** **		<0.05	***Urin/gen. tract inf.*** ***	45.46 ± 6.40	>0.05
Single	46.58 ± 6.08^a^			45.27 ± 5.36	
Married	44.96 ± 5.86^b^		Yes		
Divorced	44.37 ± 5.19^b^		No		
***Past pregnancies*** **		>0.05	***Chronic illness*** ***		<0.05
Between 1-3	45.08 ± 5.72			44.12 ± 6.49	
Between 4-6	44.73 ± 5.49		Yes	45.82 ± 5.63	
7 and above	44.03 ± 7.20		No		
***Gb longer than 24 hours*** ***			***Urinary incont*** ***		
Yes	44.45 ± 6.21	<0.05	Yes	44.91 ± 6.17	<0.05
No	45.14 ± 5.72		No	45.56 ± 5.81	

* Total number of participants. The sum of the number of participants in the subgroups may be different for some variables.

** Those with 3 or more groups in the table. were compared with One-Way Analysis of Variance (ANOVA).

*** Variables with two groups were compared with Student’s t-test.

## DISCUSSION

Women living in Mersin/Turkey and visiting local family health centers participated in this study. About 28.9% of the women included in the study experienced UI problems. Durukan et al. (2015) found the perceived urinary incontinence rate to be 21.3% in their study.^9^ Akkuş Y et al. and Pınar G et al. (2016) found this rate to be 86% in women applying to the gynecology outpatient clinic.^2^ In our study, the frequency of UI increased in advanced ages and in women with a BMI of 30 and above (52.7%). Our findings are similar to those in the literature.^10^.^11^

Despite the fear of others noticing the problem, the rate of seeking help from a doctor was low in women affected by UI. In many studies. it has been observed that the rate of seeking help is low for people with UI.^2^,^12^,^13^ Patients with mild UI can be treated with pelvic floor function exercise or with minimally invasive surgery such as transvaginal tension-free mid-urethral banding.^14^ These results suggest that women are hesitant to seek help from a health institution because UI is a urogenital health problem.

In our study, the prevalence of UI was higher in menopausal women than in non-menopausal women. The decrease in estrogen during menopause causes regression in the connective tissue, vascular and mucosal structure of the urogenital area and therefore the urethral sphincter. In menopause. the urethra may shorten by 1-2 cm. Also, this often results in muscle weakness as well as delayed re-repair of the bladder and muscles responsible for urinary excretion.^15^

We found no significant difference between the prevalence of UI in those who underwent episiotomy or perineal repair for vaginal delivery and those who did not. However, a significant difference was found in the prevalence of UI in those who had a postpartum infection. It appears that episiotomy does not protect against urinary incontinence.^16^

UI was higher in those who had given birth by normal vaginal delivery compared to those who had given birth by CS. those who received general anesthesia compared to those who received spinal anesthesia. and those who gave birth over more than 24 hours. Excessive stretching of the nerves. muscles. fascia. and ligaments of the pelvic floor in vaginal delivery can lead to irreversible anatomical and functional changes. which may cause UI. There is a positive correlation between vaginal deliveries and urethral sphincter insufficiency; however, the effectiveness of cesarean delivery in preventing of UI is controversial because both vaginal and cesarean delivery in the second stage of labor may damage the pelvic structure. No relationship was found between epidural analgesia and postpartum UI.^17^ However. in a meta-analysis. UI was high in women received anesthesia in the delivery.^18^ Our findings are similar to studies in literature..

According to our findings, the prevalence of UI was higher among women who had had seven or more pregnancies compared to those who had had fewer, among those who had given birth three or more times compared to those who had given birth 1-2 times. Those who gave birth less than two years apart had a higher prevalence of UI than those who gave birth more than two years apart. An increasing number of pregnancies cause the levator ani muscle to stretch excessively.^19^

We found that the frequency of UI increased in women who had postpartum urinary tract infections. Other studies have shown that urinary tract infections cause an increase in UI.^20^.^21^ Our findings agree with the literature findings. In our study, the frequency of experiencing UI was higher in women who knew the exercises to prevent UI. It has been reported that UI symptoms are reduced in women who practice pelvic floor exercises.^22^ It can be said that the women participating in our study knew these exercises but did not practice them sufficiently.

Furthermore as the age range of the women increased. UIAS scores decreased. and as their education level increased, their UIAS scores increased. and the difference between them was statistically significant. Despite the increase in the frequency of UI in older ages. the decrease in attitude scores suggests that women feel more social pressure.^23^ The low level of education at older ages may explain the negative attitudes of older women toward UI.

In this study, single women’s UIAS scores were higher than those of married and divorced women. and the difference between them was statistically significant. There is insufficient literature on coping with UI according to the marital status of individuals. However, in a study on coping with UI according to sex, it was reported that women sought help with UI problems much later than men did.^24^ This is an indication of the difficulty regarding independent decision-making and implementation by married women about their own health.

We also observed that women with low education levels and chronic diseases had lower UIAS scores than those without. It is thought that this situation may be related to women’s limited health literacy levels and awareness about UI.^25^ Our findings will contribute to the literature in terms of reflecting the frequency of urinary incontinence and attitudes of women in Mersin.

### Limitations:

Since this study was limited to women who visited family health centers in Mersin province for any reason, the study results can only be generalized for this region.

## CONCLUSION

Nearly one-third of the women participating in the study had UI problems. The demographic and fertility characteristics of the women influenced the frequency of UI. In addition, it was concluded that women with UI problems did not present to a health institution for various reasons and did not receive any treatment. It was also revealed that among the women participating in the study, those with negative attitudes towards UI experienced more UI problems. It is recommended to carry out studies with wider participation to determine the attitudes of women towards UI and to plan increasing awareness, education activities for women to develop positive attitudes about UI.

## Author`s Contributions:

**NA** designed the study.

**NA and BS** collected the data.

**BS** Data analysis, interpretation and responsible for the accuracy of the work.

All authors commented on the final version of the article and have read and approved the final and revised article.
